# Genomic Characterization of the Honeybee–Probiotic Strain *Ligilactobacillus salivarius* A3iob

**DOI:** 10.3390/ani15172606

**Published:** 2025-09-05

**Authors:** Mariano Elean, Alejandro Arroyo Guerra, Leonardo Albarracin, Keita Nishiyama, Haruki Kitazawa, M. Carina Audisio, Julio Villena

**Affiliations:** 1 Laboratory of Immunobiotechnology, Reference Centre for Lactobacilli (CERELA-CONICET), Tucuman CP4000, Argentina; melean@cerela.org.ar; 2 Instituto de Investigaciones para la Industria Química (INIQUI), Consejo Nacional de Investigaciones Científicas y Técnicas (CONICET), Universidad Nacional de Salta, Salta CP4400, Argentina; arroyoguerraagustin@gmail.com (A.A.G.); carina.audisio@gmail.com (M.C.A.); 3 Laboratory of Respiratory Immunology (LaRI), Division of Animal Immunology and Omics, International Education and Research Center for Food and Agricultural Immunology (CFAI), Graduate School of Agricultural Science, Tohoku University, Sendai 980-8572, Japan; lalbarracin@tohoku.ac.jp; 4 Laboratory of Animal Food Function, Graduate School of Agricultural Science, Tohoku University, Sendai 980-8572, Japan; keita.nishiyama.a6@tohoku.ac.jp; 5 Livestock Immunology Unit, International Education and Research Center for Food Agricultural Immunology (CFAI), Graduate School of Agricultural Science, Tohoku University, Sendai 980-8572, Japan

**Keywords:** *Ligilactobacillus salivarius* A3iob, honeybee, probiotic, genomics, probiotic genes, bee health

## Abstract

Background: Previous studies have demonstrated the beneficial effects of *Ligilactobacillus salivarius* A3iob on honeybee (*Apis mellifera*) colonies’ health and honey production. The present work aimed to assess the genomic characteristics of the A3iob strain to understand its ability to improve bees’ health. Methods: The comparative genomic analysis was performed with the A3iob genome and the genomes of probiotic *L. salivarius* strains of human, porcine, and chicken origin, as well as bacteria isolated from the bees’ gut. The analysis included the examination of metabolic genes and functional genes related to adhesion, the production of bioactive compounds, the modulation of the host’s immune system, and antimicrobial substances. Genes associated with antimicrobial resistance and virulence were also analyzed. Results: In silico studies revealed that *L. salivarius* A3iob possesses genes for glycosyltransferases (GTs) from the families GT2 and GT4, like *Bombella apis* and *Bombella intestinalis*, and glycosylhydrolases (GH) from the families GH1, GH2, GH13, GH36, GH65, and GH177, similar to *Apilactobacillus kunkeei*, *Enterococcus durans*, and bifidobacteria isolated from the bee intestine. The A3iob strain also has a unique genetic profile with a high number of secretion system genes and adhesion genes, including the ones coding for the SecA2/Y2 system, the mucus-binding proteins MucBP1, MucBP2, and MucBP3, and a pilus cluster (pilA, SpaA, SpaB, and sorteaseA) that has only been described in five strains of the *L. salivarius* species and in the intestinal bee-derived strain *E. durans* EDD2, which could be involved in the successful colonization of the A3iob strain in the bee gastrointestinal tract. Additionally, *L. salivarius* A3iob showed the presence of exopolysaccharide biosynthesis clusters described in the probiotic *L. salivarius* UCC118. Genes related to oxidative stress response (thioredoxin and NrdH-redoxin systems) and the bacteriocin genes abp118A and abp118B were found in the A3iob genome. *L. salivarius* A3iob does not harbor virulence or antibiotic resistance genes. Conclusions: The genomic characterization of *L. salivarius* A3iob performed in this work provides some clues about the genetic mechanisms underlying its probiotic properties, paving the way for future research aimed at improving bees’ health and productivity in the face of environmental challenges.

## Simple Summary

*Ligilactobacillus salivarius* A3iob is a beneficial microorganism isolated from the intestinal tract of honeybees. It has been demonstrated previously that the A3oib strain can improve honeybee colonies’ health and honey production. To understand the mechanisms by which probiotic *L. salivarius* A3iob exerts its beneficial effect on bees, a study of its genome was carried out in search of genes related to intestinal colonization, production of bioactive compounds, modulation of the host’s immune system, and antimicrobial substances. The genomic characterization of the *L. salivarius* A3iob strain performed in this work provides some clues about the genetic mechanisms underlying its probiotic properties. This study improves the understanding of the interaction between this beneficial microbe and honeybees.

## 1. Introduction

Bees play a pivotal role in ecosystems and economies worldwide. Ecologically, they are essential pollinators, facilitating the reproduction of a vast array of flowering plants, including many crops crucial for human consumption [1,2]. This pollination service not only sustains biodiversity but also supports the productivity and stability of ecosystems [1,2]. Economically, bees contribute significantly to agriculture, with estimates suggesting that they are responsible for pollinating approximately one-third of global food crops. Bees are not only important conservation agents for keeping floral diversity but also contribute to the human economy through the production of foods such as honey, propolis, and royal jelly [2]. Over the past few decades, a reduction in the abundance and the health of bee colonies has been observed, which has made it necessary to investigate tools that help to preserve the health of bee colonies and maintain their numbers.

Bees face numerous challenges, including diseases caused by pathogenic agents like *Varroa destructor* and *Nosema* spp., which affect bee colonies and endanger their survival [3,4]. *V. destructor* infestation on honeybees reduces bee weight and water content, affecting adult bee size and sperm production [5,6]. *Varroa* also impairs foragers’ flight and navigation abilities, hindering resource collection vital for colony development [6]. On the other hand, nosemosis is one of the main factors associated with the weakening and loss of hives, having an even greater impact when it is associated with exposure to pesticides and nutritional stress, both of which worsen the immune response of these insects [7].

While research in this area is ongoing, early studies indicate that probiotics may hold promise as a natural and sustainable intervention to manage *Varroa* sp. infestations in honeybee colonies [8]. It was observed that the administration of a probiotic mix composed of *Lactobacillus acidophilus* LA14 and *Bifidobacterium lactis* BI-04 to the hive was associated with an enhancement in the development of the colonies, probably as a result of an improvement in the bees’ digestion by increasing the secretory activity in the intestine and the length of the villi [9]. Additionally, in the groups treated with the probiotics in combination with lactic acid, a reduction in intestinal pH was found, which was associated with a decrease in the number of potentially pathogenic spores and an increase in the number of beneficial bacteria present in the intestine [9]. Studies carried out by our group showed that *Ligilactobacillus salivarius* A3iob, isolated from the honeybee intestine, presented probiotic properties when applied to bees [10]. The administration of the A3iob strain was able to confer protection against *V. destructor* and *Nosema* spp. infections [11]. Following the application of *L. salivarius* A3iob to colonies in apiaries, a significant reduction of 50 to 80% in varroasis levels was observed compared to the control group [11]. In addition, a significant decrease in the spore levels of *Nosema* spp. was observed in the lactobacilli-treated group [11]. The ability of the A3iob strain to enhance the honeybee’s resistance to infections was translated into increased yields of honey production in bees treated with the lactic acid bacterium compared to controls [10]. More recently, we showed that *L. salivarius* A3iob improves the intestinal histology of honeybees, indicating that it establishes a beneficial interaction with the cells of the intestinal mucosa (*submitted for publication*).

Genomic analysis of probiotic strains has been shown to be of value in determining bacterial molecules involved in their beneficial effects [12,13]. Thus, considering the proven effectiveness of *L. salivarius* A3iob in field studies and the availability of its complete genome sequence [14], we aimed to carry out a genomic characterization of this probiotic strain to decipher the genetic features that could be involved in its beneficial effects on honeybees. For this purpose, a genomic comparison was carried out with other bacterial strains isolated from the bee intestine, pollen, or hives or used as probiotics for bees. In addition, the A3iob strain was compared with other probiotic strains belonging to the species *L. salivarius*, isolated from different sources, which are used to improve animal or human health. The genomic analysis focused on the evaluation of genes involved in the ability of the strain to adhere and colonize the gastrointestinal tract, the production of bioactive compounds such as vitamins or growth factors, the ability to modulate the host’s immune system, and the secretion of antimicrobial substances that inhibit the growth of pathogens as well as in the presence of genes associated with antimicrobial resistance and virulence.

## 2. Materials and Methods

### 2.1. Genomes

Genomic sequence data for different bacterial strains were obtained from the National Center for Biotechnology Information (NCBI) database [15], including the genome of *L. salivarius* A3iob that was previously sequenced [14]. A total of 30 genomes belonging to the *Bombella intestine*, *Bombella apis*, *Lactobacillus acidophilus*, *Bifidobacterium lactis*, *Apilactobacillus kunkeei*, *Bifidobacterium*, *Enterococcus durans*, *Ligilactobacillus salivarius*, *Apilactobacillus micheneri*, *Apilacobacillus timberlakei*, *Apilactobacillus quenuiae*, *Apilactobacillus apinorum*, and *Apilactobacillus waqarii* species were downloaded and used for this study. The genome annotations of the different strains are summarized in Table 1. Additionally, the A3iob strain was compared with different strains belonging to the species *L. salivarius*, which are specified in Table 2.

### 2.2. Average Amino Acid Identity Analysis

Average amino acid identity (AAI) was calculated using CompareM software (version 2.1) (https://github.com/dparks1134/CompareM, accessed on 1 December 2024). From these data, an AAI heatmap was constructed using the heatmap.2 function of the gplots R package (version 3.1.3).

### 2.3. Multilocus Sequence Analysis and 16s rRNA Analysis

A phylogenetic tree was constructed with the following housekeeping gene sequences: *parB*, *rpsB*, *pheS*, *nrdB*, *groEL*, and *ftsQ.* Housekeeping genes for multilocus sequence analysis (MLSA) were selected based on previous works [13,16]. Using a multiple alignment program (MUSCLE) (v5.3) [17], the sequences were aligned, and the tree was built from the maximum likelihood estimation statistical test [18], available in MEGAX [19]. The 16s rRNA tree was built using the same parameters in the MEGA program.

### 2.4. In Silico Analysis of Presence/Absence of Functional Genes

Genomes downloaded from GenBank were uploaded to the RAST server [20]. The characteristic sequences of the different genes were obtained from the KEGG servers [21]. These sequences were then searched in the genomes of the different strains using the blastp tool in the RAST server.

### 2.5. In Silico Analysis of Glycosyl Hydrolases and Glycosyl Transferases

The protein FASTA file of each strain was downloaded from the NCBI and then uploaded to the dbCAN3 server [22]. Heatmaps were constructed using the pheatmap package (version 1.0.12) and the R package (version 3.1.3).

### 2.6. In Silico Analysis of Antibacterial Compounds

The search for bacteriocin-encoding genes was performed using the Bagel 4 Server [23] and the blastp algorithm. Additionally, the genomic identification of antibacterial substances in the genome of *L. salivarius* A3iob was performed with antiSMASH (v7.0.0) (https://antismash.secondarymetabolites.org, accessed on 1 January 2025).

### 2.7. In Silico Analysis of Virulence and Antimicrobial Resistance Genes

Virulence genes were investigated using the VFDB Database [24]. Antimicrobial resistance genes were evaluated using ResFinder (Version. 4.7.2) (http://genepi.food.dtu.dk/resfinder/job/10wlipvkwi9egorrraw61gb9mrbo9542, accessed on 1 January 2025) and RGI (https://card.mcmaster.ca/analyze/rgi, accessed on 1 January 2025). A minimum cutoff of 50% amino acid identity and 70% similarity was used. In cases that were at the limit, each case was analyzed on a case-by-case basis.

### 2.8. Statistical Analysis

Statistical analyses were performed using the tools included in the distinct program packages.

## 3. Results

### 3.1. General Genomic Characteristics of L. salivarius A3iob

The draft genome of *L. salivarius* A3iob was sequenced using Illumina HiSeq and contains 12 contigs (114.0× coverage) as described previously [14]. The A3iob draft genome sequence has an average GC content of 32.6% and a total estimated size of 2,054,490 bp. The RAST server and Prokka program allowed the prediction of a total of 2010 coding sequences, 61 tRNAs, 20 rRNAs, and 3 noncoding RNAs (ncRNAs) in the *L. salivarius* A3iob genome. These general genomic features are similar to the *L. salivarius* strains described previously [13,16,25] and the ones used in this study for comparison.

### 3.2. Phylogeny and Average Amino Acid Identity Analysis

The search of the genomes in the NCBI database belonging to strains isolated from the bee intestine, pollen, or hive allowed us to conclude that most of the published genomes belong to the species *Apilactobacillus timberlakei* and *Apilactobacillus kunkeei*. Of note, the genome of the A3iob strain is the only one from the bee intestine belonging to the *L. salivarius* species.

**Figure 1:**
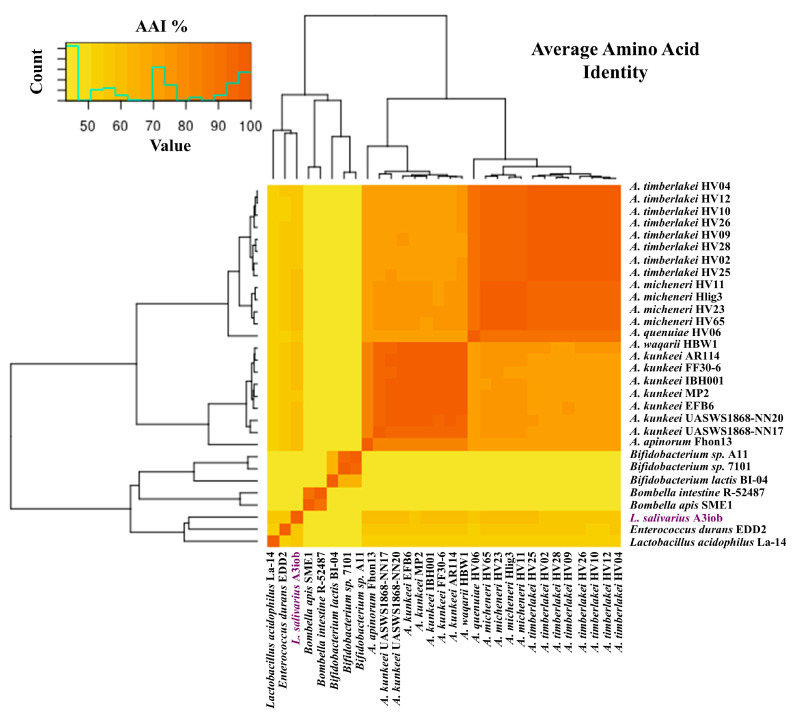
Heatmap of the Average Amino Acid Identity (AAI) for bee-related strains. Genomes of bacteria isolated from the bee intestine, pollen, or hive belonging to the genera *Apilactobacillus*, *Bombella*, *Lactobacillus*, *Bifidobacterium*, *Enterococcus*, and *Ligilactobacillus* were obtained from the National Center for Biotechnology Information (NCBI) database. Orange shades represent higher similarity between genomes, while yellow shades denote lower similarity. The precise isolation source of each strain is indicated in Table 1.

As a first comparison, we carried out an analysis of the average percentage of identity between the different bee-associated strains. As shown in Figure 1, the bacteria were grouped into two main clusters: the first one with the strains belonging to the *Apilactobacillus* genus and a second one composed of the strains belonging to the *Bombella*, *Lactobacillus*, *Bifidobacterium*, and *Ligilactobacillus* genera. The first cluster, with AAI percentages greater than 70% among the strains, presented two large subclusters of high identity, one composed of the species *A. micheneri, A. timberlakei*, and *A. quenuiae*, and a second one composed of the species *A. kunkeei, A. apinorum*, and *A. waqarii*. In the second main cluster, we found much greater variability with AAI percentages between 40 and 65% (Figure 1), a fact that was expected considering the different species included in this group.

The comparison of the 16s rRNA sequence of the A3iob strain with other probiotic strains belonging to the species *L. salivarius* demonstrated that the A3iob strain is quite distant from the other bacteria (Supplementary Figure S1A). In addition, it was observed that the *L. salivarius* strains were distributed without a relationship with their isolation source. When the MLSA was performed, the A3iob strain was grouped together with the DJ-sa-01 strain isolated from the chicken gut (Supplementary Figure S1B). Again, there was no clear relationship between the clusters and the origin, although the strains isolated from human sources were located in the same clusters.

### 3.3. Analysis of Adhesion Genes

**Figure 2:**
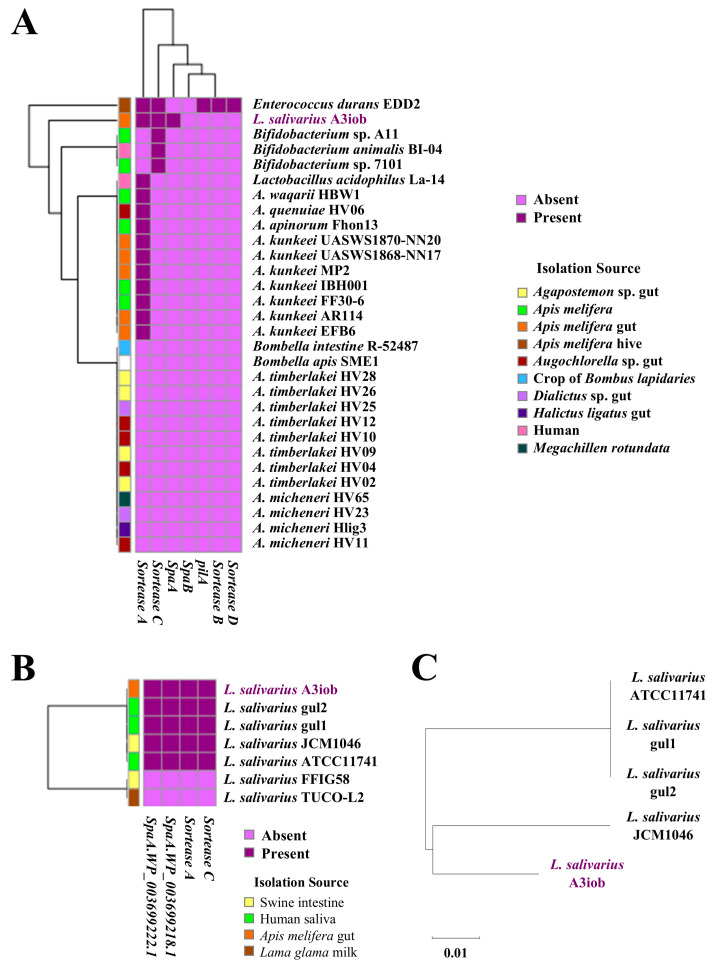
Pilus operon genes in the *Ligilactobacillus salivarius* A3iob genome compared to (**A**) genes present in the genomes of bee-related strains belonging to the genera *Apilactobacillus*, *Bombella*, *Lactobacillus*, *Bifidobacterium*, and *Enterococcus*, and (**B**) other probiotic *L. salivarius* strains. (**C**) Phylogenetic tree constructed with the pilus operon genes present in *L. salivarius* A3iob and other probiotic strains of the same species. Colored boxes indicate the presence (dark violet) or absence (light violet) of genes. In addition, the distinct isolation sources of each strain are shown with different colored boxes.

Considering the importance of the pili as adhesion structures that facilitate bacteria–cell host interactions, we studied the presence of genes coding for pili and related proteins in the different bee-associated bacteria (Figure 2A). We searched for *pilA*, *SpaA*, *SpaB*, *sorteaseA*, *sorteaseB*, *sorteaseC*, and *sorteaseD* genes. The analysis showed that *E. durans* EDD2 and *L. salivarius* A3iob had the highest number of pili-associated genes.

Both strains presented the genes *sorteaseA* and *sorteaceC*; however, *SpaA* was only present in A3iob, whereas *pilA*, *sorteaseB*, and *sorteaseC* were only present in the EDD2 strain. In the *Bifidobacterium* strains, only the *sorteaseC* gene was present, while in *L. acidophilus* La-14 and in the strains belonging to *A. kunkeei*, *A. quenuaei*, and *A. waqarii* species, only *sorteaseA* was found (Figure 2A). All of the strains belonging to *A. timberlakei*, *A. micheneri*, *B. intestini*, and *B. apis* species lacked all of these pilin protein-encoding genes. When A3iob was compared with other *L. salivarius* strains, we found the same profile in the strains Gul1, Gul2, JCM1046, and ATCC11741 (Figure 2B). On the contrary, none of these genes were present in *L. salivarius* FFIG58 and *L. salivarius* TUCO-L2. The comparison of the pili operon sequences in *L. salivarius* A3iob with the sequences described in four strains of the same species [16] revealed three different pili: one shared by ATCC11741, gul1, and gul2 strains, another for *L. salivarius* JCM1046, and a third one for the A3iob strain (Figure 2C).

**Figure 3:**
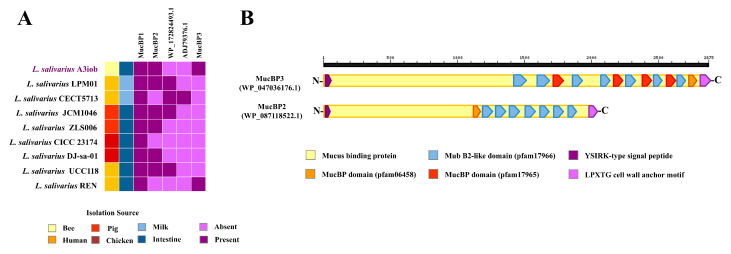
Mucus-binding proteins (MucBP) genes in the *Ligilactobacillus salivarius* A3iob genome. (**A**) Genomic comparison of the MucBP genes from the *L. salivarius* A3iob genome with those present in the genomes of other probiotic strains of the same species. Colored boxes indicate the presence (dark violet) or absence (light violet) of genes. In addition, the distinct isolation sources of each strain are shown with different colored boxes. (**B**) Schematic representation of the domains present in MucBP2 and McBP3 in the genome of *L. salivarius* A3iob. Colored boxes indicate the distinct domains found in both proteins.

We searched for the presence of genes encoding for mucin-binding proteins (MucBPs), which play a key role in adhesion to gastrointestinal mucin, in the *L. salivarius* A3iob genome. Previously, we described the presence of three MucBPs in the genome of *L. salivarius* FFIG58 [26] and TUCO-L2 [13], which were designated as MucBP1, MucBP2, and MucBP3. We found the genes MucBP1, MucBP2, and MucBP3 in the A3iob strain (Figure 3A). The MucBP1 gene is present in all *L. salivarius* strains, while MucBP2 (WP_047036176.1) and MucBP3 (WP_087118522.1), which are proteins of different lengths and with different numbers of MucBP (pfam06458), MubB2-like (pfam 17966), and MucBP (pfam17965) domains (Figure 3B), were described in only a few strains of this species [13,26]. Up to seven different MucBP orthologs were found in the pangenome of *L. salivarius* [25], including WP_172824493.1 and ADJ79376.1, which are present in the genome of probiotic strains [26]. Neither of these two genes was found in the genome of *L. salivarius* A3iob (Figure 3A).

**Figure 4:**
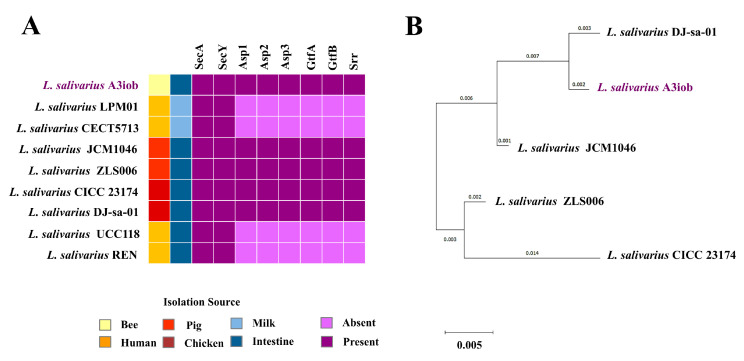
SecA/SecY secretion system genes in the *Ligilactobacillus salivarius* A3iob genome. (**A**) Genomic comparison of the SecA/SecY secretion system genes from *L. salivarius* A3iob with those present in the genomes of other probiotic strains of the same species. Colored boxes indicate the presence (dark violet) or absence (light violet) of genes. In addition, the distinct isolation sources of each strain are shown with different colored boxes. (**B**) Phylogenetic tree constructed with the SecA/SecY secretion system genes present in *L. salivarius* A3iob and other probiotic strains of the same species.

Additionally, we investigated the presence of the SecA/SecY secretion system genes in the genomes of *L. salivarius* strains. The A3iob, JCM1046, ZLS006, CICC23174, and DJ-sa-01 strains presented the complete cluster composed of the genes *SecA*, *SecY*, *Asp1*, *Asp2*, *Asp3*, *GtfA*, *GtfB*, and *Srr* (Figure 4A). The strains LPM01, CECT5713, UCC118, and REN only have the genes *SecA* and *SecY.* A phylogenetic tree was constructed with the SecA/SecY genes, and it was observed that the A3iob was grouped close to the DJ-sa-01 strain and separated from the other *L. salivarius* strains (Figure 4B).

### 3.4. Analysis of Genes Related to Exopolysaccharide Production, Glycosyl Hydrolases, and Glycosyl Transferases

**Figure 5:**
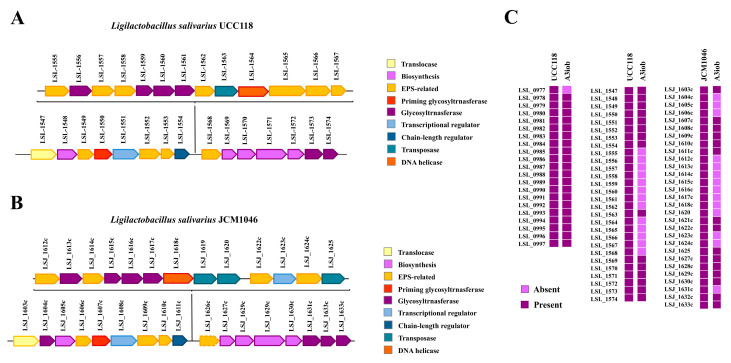
Genes involved in EPS biosynthesis in the *Ligilactobacillus salivarius* A3iob genome. Comparison of the biosynthetic cluster for EPS between the *L. salivarius* A3iob and (**A**) *L. salivarius* UCC118 or (**B**) *L. salivarius* JCM1046. Colored boxes indicate the function of genes during EPS biosynthesis. (**C**) Genomic comparison of the genes involved in EPS biosynthesis from *L. salivarius* A3iob with those present in the genomes of the probiotic strains UCC118 and JCM1046. EPS1 (LSL_0977 to LSL_0997), EPS2 (LSL_1547 to LSL_1574), and EPS3 (LSJ_1603c to LSJ_1633c) were studied. Colored boxes indicate the presence (dark violet) or absence (light violet) of genes.

We searched for the presence of genes encoding proteins involved in exopolysaccharides (EPS) biosynthesis in *L. salivarius* A3iob. For this purpose, we used as a reference the EPS clusters described in the strains UCC118 [16] and JCM1046 [27]. *L. salivarius* UCC118 possesses two EPS gene clusters, the EPS1 containing 20 genes (LSL_0977 to LSL_0997) and the EPS2 containing 27 genes (LSL_1547 to LSL_1574, Figure 5A), while *L. salivarius* JCM1046 has the EPS3 gene cluster containing 28 genes (LSJ_1603c to LSJ_1633c, Figure 5B). All of the genes of the EPS1 cluster were detected in the genome of the A3iob strain except for the LSL_0977 gene (Figure 5C). On the other hand, the conserved genes in the EPS2 and EPS3 clusters [16,27] were detected in the A3iob genome (Figure 5C). The genes LSL_1555 to LSL_1562 and LSL_1564 to LSL_1568 of the EPS2 cluster, which code for distinct glycosyltransferases, were absent in *L. salivarius* A3iob. In addition, the genes LSJ_1604c to LSJ_1606c, LSJ_1612c to LSJ_1620, LSJ_1623c, LSJ_1624c, and LSJ_1631c, most of which code for glycosyltransferases, were also absent in the A3iob genome (Figure 5C). Different types of glycosyltransferases in the genomes of microorganisms able to synthesize EPS can modify the structure and functionality of the molecule. In fact, it was demonstrated that distinct glycosyltransferases among 42 *L. salivarius* strains allowed for the production of variable structures of EPS that affected their interactions with biotic environmental factors [16].

**Figure 6:**
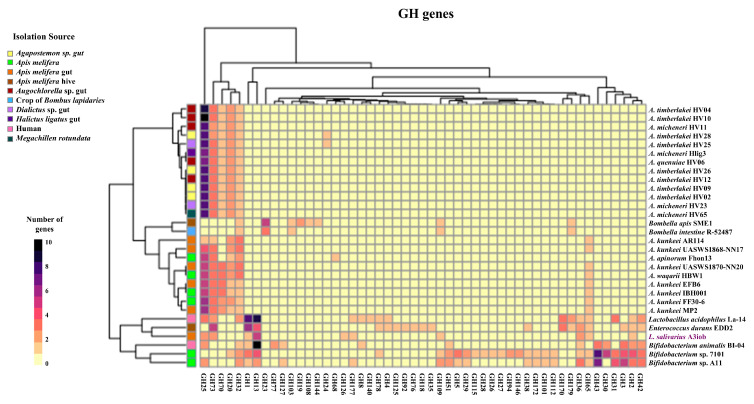
Distribution of glycosyl hydrolase (GH) genes in the *Ligilactobacillus salivarius* A3iob genome compared with the genomes of bacteria isolated from the bee intestine, pollen, or hive belonging to the genera *Apilactobacillus*, *Bombella*, *Lactobacillus*, *Bifidobacterium*, and *Enterococcus*. Colored boxes indicate the number of genes in each GH family from the highest value (black) to the lowest value (yellow). In addition, the distinct isolation sources of each strain are shown with different colored boxes.

**Figure 7:**
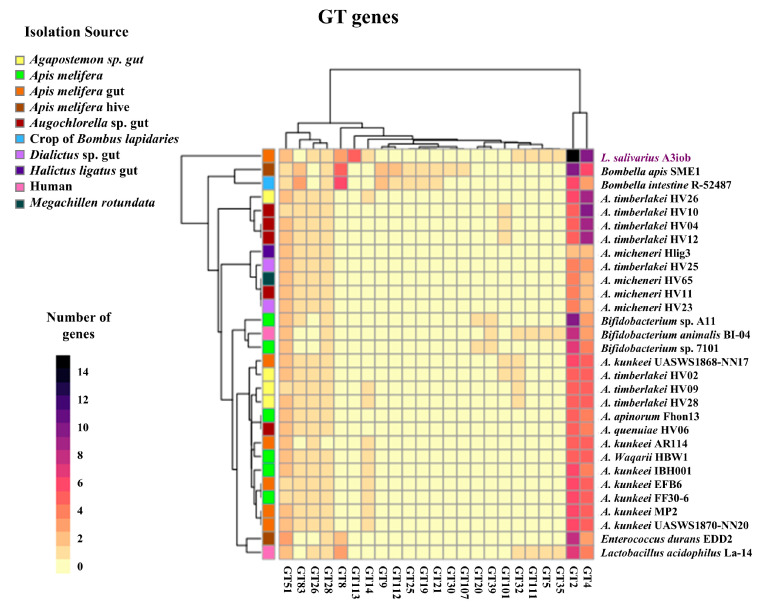
Distribution of glycosyl transferase (GT) genes in the *Ligilactobacillus salivarius* A3iob genome compared with the genomes of bacteria isolated from the bee intestine, pollen, or hive belonging to the genera *Apilactobacillus*, *Bombella*, *Lactobacillus*, *Bifidobacterium*, and *Enterococcus*. Colored boxes indicate the number of genes in each GT family from the highest value (black) to the lowest value (yellow). In addition, the distinct isolation sources of each strain are shown with different colored boxes.

We analyzed the presence of genes for glycosyl hydrolases (GH) and glycosyl transferases (GT) and established a comparison between the A3iob strain and other bee-related strains (Figure 6 and Figure 7) and with probiotic strains belonging to the *L. salivarius* species (Supplementary Figure S2). The strains belonging to the species *A. timberleakei*, *A. micheneri*, and *A. quenulae* presented a similar profile of GH, characterized by the presence of the GH20, GH25, GH32, GH70, and GH73 families (Figure 6). In this group, the strains *A. timberlakei* HV04 and *A. timberlakei* HV10 stood out as those with the highest number of GH25 genes. *B. apis* SME1 and *B. intestini* R-52487 were markedly different from this group because they had genes coding for GH23, GH103, GH109, and GH170, while they lacked the genes GH20, GH25, GH70, and GH73. The *A. kunkeei*, *A. apinorum*, and *A. waqarii* strains were grouped together and were different from the other strains of the genus *Apilactobacillus*, having a lower number of genes for GH25 and a higher number of genes for GH65. The exception was *A. apinorum* Fhon13, which does not have GH65 but presents GH68 (Figure 6). *L. acidophilus* La-14 and *E. durans* EDD2 presented a high number of genes of the GH1 and GH13 families. On the other hand, the strains belonging to the *Bifidobacterium* genus had an intermediate number of genes for GH2, GH3, and GH42, and a high number of genes for GH13 and GH43. The A3iob strain had quite a different profile compared with the other bacteria used for the genomic analysis, presenting a high number of genes coding for GH13 and a moderate number of genes for GH1, GH2, GH25, GH36, GH65, GH73, GH109, and GH177 families. *L. salivarius* A3iob was also the only strain with a gene for GH126 (Figure 6). When the GHs of A3iob were compared with different *L. salivarius* strains, a similar profile was found, except for GH32, which was absent in the bee strain (Supplementary Figure S2A).

The analysis of the genes for GTs revealed that all of the studied strains had the genes GT2, GT4, and GT51, while the gene GT28 was absent only in *A. kunkeei* AR114 (Figure 7). Interestingly, the number of genes of the GT2 and GT4 families was higher in *L. salivarius* A3iob compared to the other strains. GT5, GT35, and GT111 were present only in the A3iob strain and in *B. animalis* BI-04 and *L. acidophilus* La-14. In addition, *L. salivarius* A3iob was the only strain with GT113 genes, while the GT83 family that was detected in the genomes of most of the bee-related bacteria was not present in A3iob (Figure 7). When the strain A3iob was compared with other *L. salivarius* strains, it stood out for having the highest number of copies for GT2, GT4, and GT113 genes (Supplementary Figure S2B).

### 3.5. Analysis of Genes for Vitamin Production and Antioxidant Systems

The comparison of the percentage of genes across different metabolic categories for *L. salivarius* A3iob with other strains of the same species revealed a similar profile (Supplementary Figure S3A). However, the number of genes involved in the metabolism of carbohydrates and proteins, as well as in amino acids and derivatives in *L. salivarius* A3iob, was higher than in the other strains of the same species (Supplementary Figure S3B). We found that the number of genes related to the production of vitamins and cofactors was the same in all of the strains (Supplementary Figure S3B). Subsequently, we evaluated the presence or absence of specific genes involved in the production of B-group vitamins in *L. salivarius* A3iob. No genes were detected for the production of niacin (vitamin B3) (Supplementary Figure S4A), pantothenic acid (vitamin B5) (Supplementary Figure S4B), or vitamin B12. The *ThiT* gene for the thiamine (vitamin B1) uptake system and the *ThiD* gene, which encodes the enzyme that catalyzes the conversion of hydroxymethyl pyrimidine phosphate to hydroxymethyl pyrimidine pyrophosphate, were detected (Supplementary Figure S5). The A3iob strain did not have genes for the de novo synthesis of biotin (vitamin B7) but had genes for its transport (*BioY*) (Supplementary Figure S6A). For riboflavin (vitamin B2), genes involved in the uptake of this vitamin from the medium (*RibT* and *RibU*) and for its transformation to FAD (*RibCF*) were detected, but not for de novo synthesis (Supplementary Figure S6B). In the case of pyridoxine (vitamin B6) metabolism, only the *dxs*, which codes for a d-glyceraldehyde-3-phosphate transporter, and the *gapA* and *PdxK* genes, which code for the enzymes of the first and last steps of the pathway, were found in the A3iob genome (Supplementary Figure S7). When folate (vitamin B9) metabolism was investigated, the *FolT* gene that allows folate to be incorporated from the environment and the genes *FolC*, *FolA*, and *FolD* from the de novo late step were detected (Supplementary Figure S8). The same gene profile was found in the genomes of *L. salivarius* JCM1046, ZLS006, UCC118, and REN for vitamin biosynthesis. Therefore, all of these strains would be incapable of producing B-group vitamins and reliant on the uptake system for growth.

In addition, we detected in the A3iob genome the presence of three *trxA* genes for thioredoxin and a gene for thioredoxin reductase (protein IDs PWG53332.1, PWG50881.1, and PWG53839.1, respectively). The *trxA* genes had 61, 61, and 39% identity (80, 77, and 63% similarity) with the genes previously described in *L. casei* [28]. We also found the presence of an NrdH-redoxin system independent of glutathione, similar to that described in *E. coli* [29].

### 3.6. Analysis of Antimicrobial Genes

Natural products with antimicrobial activities are an interesting alternative to combat infections. For lactic acid bacteria, it has been shown that bacteriocins have the potential to be used to prevent or treat infections [30]. Then, we evaluated the genome of *L. salivarius* A3iob utilizing the BAGEL 4 platform and blastp to identify putative antimicrobial substances. The analysis indicated the presence of the genes *abp118A* (PWG51651.1) and *abp118B* (PWG51650.1), which encode for the chain A and chain B of the Abp118 bacteriocin, respectively. The *abp118A* gene is identical to that previously described for *L. salivarius* UCC118 (100% identity) [31], whereas the *abp118B* sequence shares 98.5% identity. Upstream of both genes, a putative immunity gene (*abpiM)* was found. The absence of other antimicrobial compounds distinct from bacteriocins in the genome of the A3iob strain was confirmed with the antiSMASH database.

### 3.7. Analysis of Antimicrobial Resistance and Virulence Genes

The genomes of *L. salivarius* A3iob, as well as other strains isolated from the bee gut or environment, were investigated to search for the presence of genes potentially related to negative effects. The evaluation of antimicrobial resistance genes detected the presence of the gene *rbpO* in the genomes of the *Bifidobacterium* species, which confers resistance to rifamycin (Supplementary Table S1). The gene *tetW* that codes for a tetracycline-resistant ribosomal protection protein was found only in *B. animalis* BI04, while the gene ermB associated with resistance to macrolide, lincosamide, and streptogramin antibiotics was found only in the genome of *A. kunkeei* AR114. *E. durans* EDD2 harbors the gene *AAC(6′)-Iih* that confers resistance to aminoglycosides (Supplementary Table S1). Of note, no antimicrobial resistance genes were observed in the genome of *L. salivarius* A3iob. The search in databases indicated the presence of *carB* coding for a carbamoyl phosphate synthase and *gndA* coding for NADP-dependent phosphogluconate dehydrogenase in the genomes of bifidobacteria strains A11 and 7101, respectively (Supplementary Table S2). Although informatics analysis indicated those genes as potential virulence factors, there is no literature available describing bifidobacteria inducing detrimental effects on the host through these proteins. On the other hand, the hasC gene that was described as a virulence factor in pathogenic streptococci [32] was found in the genome of *E. durans* EDD2 (Supplementary Table S2). No genes related to virulence were found in the genome of the A3iob strain.

## 4. Discussion

**Figure 8:**
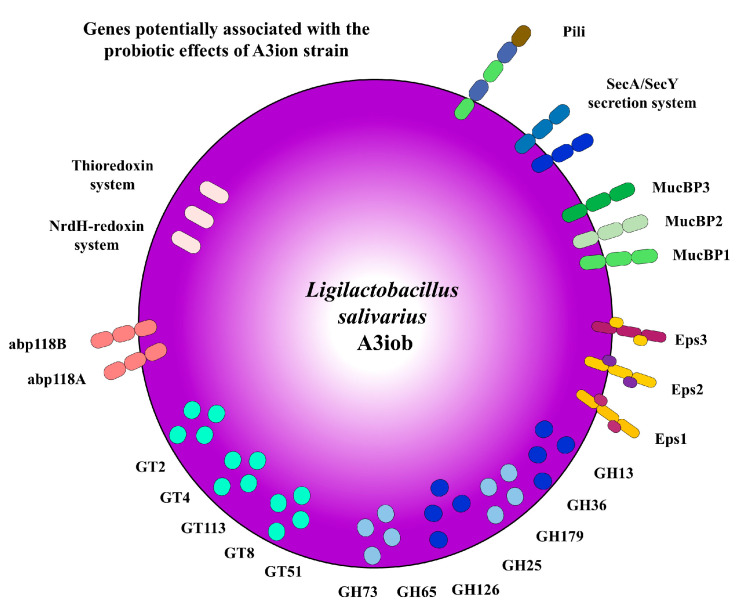
Schematic representation of *Ligilactobacillus salivarius* A3iob highlighting the genes/proteins that would be involved in its probiotic activities in honeybees, including metabolic (specific GH and GT families), ROS detoxification (thioredoxin and NrdH-redoxin systems), antimicrobial (Abp118 bacteriocin), and adhesion (EPS, pili, MucB proteins, and SecA/SecY system) genes.

There are several factors contributing to the decline in honeybee populations, including the use of pesticides [33], climate change [34], monoculture farming practices [35], habitat loss due to urbanization [1], and pathogens like *Varroa* and *Nosema* [11]. Efforts are being made to address these issues through sustainable farming practices, promoting bee-friendly habitats, reducing pesticide exposure, and supporting bee health research [36,37,38,39]. Some studies have emphasized the role of gut microbiota in bee nutrition and pathogen resistance, underscoring the potential benefits of probiotic interventions in maintaining a healthy gut microbiome in bees [39]. In this regard, we have previously shown that the administration of *L. salivarius* A3iob to colonies in apiaries improves health and resistance to infections in honeybees, leading to enhanced honey production [10,11]. Although the benefits of A3iob administration have been proven, the mechanism(s) underlying its health-promoting capacities have not been investigated in depth. We recently demonstrated that *L. salivarius* A3iob improves honeybees’ intestinal histology, indicating that this bacterium is able to establish a beneficial interaction with the cells of the intestinal mucosa (submitted for publication). Therefore, in this work, we performed a comparative genomic analysis using the A3iob genome, the genome of *L. salivarius* strains with probiotic properties, as well as the genomes of different species of bacteria isolated from the bee gut or environment to describe the set of genes that would be associated with its beneficial effects (Figure 8). We focused in particular on the genes that could explain the ability of *L. salivarius* A3iob to interact with host cells or with antagonistic action against pathogens. We detected in the A3iob genome the presence of genes that would be involved in the survival in the bee’s gut, efficient intestinal colonization, the inhibition of pathogens, and the regulation of the redox balance.

The genomic comparison of *L. salivarius* A3iob with other strains of the same species revealed no marked differences in the genes involved in metabolic capacities, except for the slightly higher number of genes involved in carbohydrate metabolism. This agrees with the slight quantitative differences in the numbers of GH and GT genes found in *L. salivarius* A3iob compared to the other strains. Bees consume floral nectar that provides sugars and pollen that provides amino acids, lipids, and vitamins. The complex molecules of the bee diet, like the pectin of pollen’s cell wall, can be assimilated by the action of GT, GH, polysaccharide lyases, carbohydrate esterases, and carbohydrate-binding modules provided by the intestinal microbiota [40]. It has been shown that *Gilliamella* and *Bifidobacterium* are implicated as the primary degraders of complex sugar molecules, while *Snodgrassella* and *Lactobacillus* play little or no role in polysaccharide digestion. However, lactobacilli can proliferate in the bee intestine due to their abilities to use simpler sugar molecules consumed by the bee or produced through polysaccharide digestion [40]. It has been shown that *Lactobacillus* spp. Firm-4 and Firm-5 from the bee intestine possess the capacity to produce GH31 (α-glucosides, α-xylosides, and α-galactosides) and GH78 (rhamnogalacturonases), while Firm-5 also produces GH29 [40,41]. None of these GH families was detected in the genome of *L. salivarius* A3iob. In contrast, this strain has a high number of genes for GH13 and was the only one with GH126 among the bacteria evaluated in this work. The presence of GH13 genes has been described in the genomes of *Lactobacillus melliventris* Hma8, *Lactobacillus kimbladii* Hma2, and *Lactobacillus kullabergensis* Biut2 isolated from the bee gut [40]. Interestingly, among more than 200 genomes of bee gut bacteria, including *Apibacter* spp., *Bartonella* spp., *Bifidobacterium* spp., *Lactobacillus* spp., *Gilliamella* spp., *Parasaccharibacter* spp., *Serratia* spp., and *Snodgrassella* spp., only *Gilliamella apicola* P46G possessed a GH126 gene [40]. In addition, the genome of A3iob harbors genes from the GH25 family, which are abundant in the genomes of the bee intestine-associated strains of the species *Lactobacillus apinorum* and *L. kunkeei*, as well as genes from the GH73 family that were described to be present in the genomes of *Lactobacillus mellis* and *L. kunkeei* isolated from the bee gut [40]. A3iob also has a high number of GT2 and GT4 genes similar to *Lactobacillus mellifer* Bin4, *L. mellis* Hon2, *L. melliventris* Hma8, *Lactobacillus kimbladii* Hma2, *L. kullabergensis* Biut2, and *L. kunkeei* AR114 from the bee gut [40]. Additionally, we detected genes for GT8 and GT51 in the genome of *L. salivarius* A3iob, which are abundant in the genomes of bacteria of different species isolated from the bee intestine, including *L. mellis* Hon2, *L. melliventris* Hma8, *L. apis* Hma11, and *L. mellifer* Bin4 [40]. These results suggest that the A3iob strain possesses a set of genes that would allow it to take advantage of carbohydrate sources in the bee’s intestine, allowing its growth and colonization, which is in line with the origin of the strain [8,14].

Adhesion factors play a crucial role in the colonization of bee intestines by beneficial bacteria, allowing them to attach to the intestinal surface and establish a stable presence in the host [42]. Then, adhesion factors are key molecules contributing to the establishment of symbiotic relationships between beneficial bacteria and bees, impacting health and performance [43]. Herein, we have demonstrated the presence of genes encoding for proteins of pili, the SecA/SecY secretion system, and MucBPs in the genome of *L. salivarius* A3iob. Among the bacteria studied, the A3iob strain stood out for presenting the greatest number of genes associated with adhesion, including *sorteaseA*, *sorteaseC*, *SpaA*, three MucBP, and a complete SecA2/SecY2 secretion system.

The presence of pili in probiotic *L. salivarius* strains has not been evaluated in depth. However, genomic analysis predicted the presence of pilus biosynthetic gene clusters in a few strains of this species. Previous studies have shown that among 43 genomes, only five *L. salivarius* strains harbored genes involved in pilus biosynthesis [16]. Herein, we describe the presence of a pilus gene cluster in the genome of *L. salivarius* A3iob. The presence of this adhesion factor could allow the A3iob strain to efficiently colonize the bee gut, as has been suggested for *E. durans* EDD2, which was isolated from freshly collected pollen granules from beehives [44]. On the other hand, MucBPs contain variable numbers of mub repeats that mediate the adhesion of bacteria to mucin glycans. Seven MucBP orthologous have been described in *L. salivarius* species [16,25,27]. *L. salivarius* A3iob possesses the MucBP1 found in all strains of the same species, as well as MucBP2 and MucBP3 detected in the human probiotic strains *L. salivarius* UCC118 and REN, respectively. The presence of MucBP2 has been observed in the genome of the porcine probiotic strains *L. salivarius* JCM1046 and ZLS006 [25], while MucBP3 was detected in the immunomodulatory *L. salivarius* FFIG58 porcine strain [26]. The SecA2/SecY2 cluster encodes two glycosyltransferases (*GtfA* and *GtfB*), three chaperones (*Asp1*, *Asp2*, and *Asp3*), a membrane translocation complex (*SecY2*), and a motor protein (*SecA2*) [45]. This group of genes mediates the glycosylation and exportation of the glycosylated srr adhesins that have been predicted to mediate the adhesion of *L. salivarius* strains to the gut [16,27]. Interestingly, it was shown that the genomes of strains isolated from pigs and chickens have the SecA2-SecY2 system, while it is not present in *L. salivarius* genomes of human origin [25]. We demonstrate herein that, similarly to strains isolated from animals, *L. salivarius* A3iob of bee origin possesses the SecA2/SecY2 system. In addition, we detected a conserved organization in the srr protein of A3iob, characterized by the presence of a C-terminus LPXTG cell wall-anchoring motif, highly repeated serines, an adhesion AST domain, and an N-terminus KxYKxGKxW signal peptide, as has previously been described [46]. This genetic repertoire of adhesion factors could help *L. salivarius* A3iob to carry out successful colonization of the bee gut and allow it to compete against other pathogenic and non-pathogenic bacterial strains. Colonization also allows for interaction between beneficial microbes and the host immune system, and therefore, genes associated with intestinal colonization could have a role in immunomodulation. Investigating whether some of these genes that mediate intestinal colonization of the A3iob strain also participate in immunomodulation is an interesting topic for future investigations.

Some EPS produced by bacteria have been shown to be involved in the improvement of intestinal health and protection against pathogens [47,48]. Interestingly, it was demonstrated that purified EPS from *A. kunkeei* K1.10 and *Latilactobacillus curvatus* Kar.9b, isolated from the microbiota of honeybees, have the capacity to inhibit the formation of biofilm by *Streptococcus mutans* [49]. Similarly, the EPS from the bee gut-resident *Enterococcus* sp. BE11 was able to inhibit the growth of the pathogens *Streptococcus agalactiae*, and *Staphylococcus epidermidis* [50]. On the other hand, EPS obtained from *L. salivarius* BIS312 and BIS722 were capable of inhibiting biofilm formation by pathogenic *Enterococcus faecalis*, *Staphylococcus aureus*, and *Escherichia coli* [51]. The study of the genes involved in EPS production in *L. salivarius* A3iob demonstrated the presence of two EPS clusters. One of the clusters was similar to that described in the UCC118 strain, while the second cluster was homologous to that reported in *L. salivarius* JCM1046 [16,25]. Although it has been shown that *L. salivarius* UCC118 produces low levels of EPS, this molecule has been associated with adhesion and immunomodulation [16]. These studies allow us to speculate that the EPS of *L. salivarius* A3iob could be involved in its probiotic activities in bees. Producing, purifying, and carrying out chemical and functional characterizations of the A3iob strain’s EPS is also an interesting topic for future research.

The production of bacteriocins by probiotics represents an important mechanism by which they can modulate the composition of the microbiota and promote a healthier microbial balance in the gut [30]. Some bacteriocins have a narrow spectrum of activity, meaning they selectively target specific bacterial species or strains while leaving beneficial bacteria unaffected [52]. This specificity allows probiotics to target harmful bacteria without harming beneficial ones. Then, the use of bacteriocin-producing probiotics to inhibit pathogens in bees has emerged as an interesting alternative. For instance, it has been shown that *E. durans* EDD2 has inhibitory activity against *Paenibacillus larvae* and that this effect was associated with genes coding for enterocin L50A/L50B and enterocin P-like bacteriocins [44]. Similarly, inhibitory activity against *Melissococcus plutonius* was found in the culture supernatant of *L. kunkeei* FF30-6 that was attributed to the presence of bacteriocins [53]. Here, we found the *abp118A* and *abp118B* genes in the A3iob genome, which were similar to the previously characterized two-component Abp118 bacteriocin in *L. salivarius* UCC118 [31]. It was shown that the UCC118 has anti-infective activity due to the production of the broad-spectrum class IIb bacteriocin Abp118. *L. salivarius* UCC118 protects against *Listeria monocytogenes* infection in mice due to the production of the bacteriocin Abp118 [54]. It has also been reported that the Abp118 bacteriocin has inhibitory effects on other Gram-positive bacteria like *Bacillus coagulans* [55], although it has not been described as having an effect on *V. destructor* and *Nosema* spp. These results allow us to speculate that the Abp118 bacteriocin would be involved in the ability of the A3iob strain to protect honeybees against Gram-positive pathogens, although we cannot associate it with the beneficial effects of *L. salivarius* A3iob against *V. destructor* and *Nosema* spp. described in our previous in vivo studies [10,11]. Interestingly, it has been reported that *L. salivarius* UCC118 significantly upregulated Abp118 genes upon exposure to the intestinal epithelial cells Caco-2 [56], suggesting that the enhanced bacteriocin production may aid colonization of the bacteriocin producer, conferring the lactobacilli a competitive advantage.

The generation of reactive oxygen species (ROS), including superoxide, hydrogen peroxide, and hydroxyl radicals, plays a significant role in the process of senescence in insects. ROS contribute to age-related cellular damage, mitochondrial dysfunction, and immune dysregulation, ultimately leading to physiological decline and decreased lifespan in bees [57]. Recent findings indicate that queens exhibit a longer lifespan compared to workers, and this fact was attributed to their possession of a healthier microbiota with enhanced capacity for ROS detoxification [57]. Moreover, nutrition was a significant determinant underlying this disparity [57]. Probiotics have emerged as a possible intervention to confer protection against oxidative stress since these beneficial bacteria can exert antioxidative effects directly by scavenging free radicals and chelating metal ions or indirectly by regulating host enzymes and modulating the gut microbiota [58]. The study of the oxidative stress response genes within the genome of the A3iob strain revealed the presence of genes encoding for pyruvate oxidase *pox* and lactate oxidase *lox*, while genes for NADH oxidases *nox* and catalases were absent. Notably, a thioredoxin system was detected in the A3iob genome, exhibiting homology to the system described for the probiotic bacterium *Lacticaseibacillus casei* Shirota [28]. Considering the established role of thioredoxins in conferring resistance to oxidative stress in certain lactic acid bacteria [59,60,61], these genes may provide an adaptive advantage to *L. salivarius* A3iob, potentially facilitating symbiotic interactions with the bee. Additionally, we found an NrdHIEF operon similar to that described in *E. coli* [29], which would also have thioredoxin activity. The presence of these systems may contribute to the maintenance of a healthy redox balance at the intestinal level in bees supplemented with the probiotic A3iob strain.

The intestinal microbiota not only plays a fundamental role in the inhibition of pathogens’ growth and the stimulation of the host’s defenses, but it can also exert beneficial nutritional effects, such as producing vitamins and metabolites that bees cannot produce on their own. Some studies have reported beneficial effects of B-group vitamins supplementation, including the enhancement of bee health and colony performance [62,63]. Furthermore, it was seen that vitamin supplementation improves the resistance of bees to pathogens such as *Nosema* and to viral infections such as deformed wing virus [62,63]. Studies also revealed that a combination of amino acids and vitamins led to an enhancement in hygienic behavior in bees, thereby enabling better pathogen resistance [64]. Considering this background, we studied the presence of genes for vitamin production in *L. salivarius* A3iob. We found that the A3iob strain would not be able to perform de novo synthesis of group B vitamins, presenting only genes for their import, bioconversion, and utilization. These results allow us to rule out the direct production of vitamins as a mechanism for the beneficial effects of this strain. However, the possibility that the A3iob strain may induce modifications in the bee microbiota and indirectly promote increased vitamin production should be investigated. This hypothesis is supported by the presence of genes for host–cell adhesion and bacteriocin production, which can enable *L. salivarius* A3iob to compete with and inhibit the growth of other intestinal bacteria, modifying the bee microbiota.

## 5. Conclusions

In summary, we have demonstrated herein that the genome of *L. salivarius* A3iob possesses a set of diverse genes that together could explain the ability of this beneficial bacterium to establish a symbiotic relationship with honeybees, improve their resistance to infections, and thus promote better performance in honey production. Among these genes, metabolic (specific GH and GT), ROS detoxification (thioredoxin and NrdH-redoxin systems), antimicrobial (Abp118 bacteriocin), and adhesion (EPS, pili, MucB proteins, and SecA/SecY system) genes (Figure 8) are interesting candidates to explain the beneficial effects of the A3iob strain. Further studies are needed to demonstrate the role of each of these genes in the probiotic properties of *L. salivarius* A3iob, for example, through the development of mutant strains. The genomic characterization of *L. salivarius* A3iob performed in this work provides some clues about the genetic mechanisms underlying its probiotic properties, paving the way for future research aimed at improving bees’ health and productivity in the face of environmental challenges.

## Figures and Tables

**Table 1:** Genomes of bacterial strains used in this work. The genomes were obtained from the National Center for Biotechnology Information (NCBI) database.

Species	Strain	GenBank	Isolation Source
*Bombella intestine*	R-52487	GCA_002003665.1	Crop of a bumblebee *B. lapidaries*
*Bombella apis*	SME1	GCA_009362775.1	Hive *Apis melifera*
*Lactobacillus acidophilus*	La-14	GCA_000389675.2	Human
*Bifidobacterium lactis*	BI-04	GCA_000022705.1	Fecal sample from a healthy adult
*Apilactobacillus kunkeei*	EFB6	GCA_000687335.1	*Apis mellifera* gut
*Apilactobacillus kunkeei*	UASWS1868-NN17	GCA_005930975.1	*Apis melliera* gut
*Apilactobacillus kunkeei*	UASWS1870-NN20	GCA_005930935.1	*Apis mellifera* gut
*Apilactobacillus kunkeei*	AR114	GCA_000830375.1	*Apis mellifera* gut
*Apilactobacillus kunkeei*	MP2	GCA_001314945.1	Chilean honeybee gut
*Apilactobacillus kunkeei*	FF30-6	GCA_001949975.2	*Apis mellifera*
*Apilactobacillus kunkeei*	IBH001	GCA_026428215.1	*Apis mellifera*
*Bifidobacterium sp.*	A11	GCA_000499185.1	*Apis mellifera*
*Bifidobacterium sp.*	7101	GCA_000499285.1	*Apis mellifera*
*Enterococcus durans*	EDD2	GCA_010974995.1	Pollen from *A. melifera* beehives
*Ligilactobacillus salivarius*	A3iob	GCA_003129685.1	*Apis mellifera* gut
*Apilactobacillus micheneri*	Hlig3	GCA_002993975.1	*Halictus ligatus* gut
*Apilactobacillus micheneri*	HV_11	GCA_006493545.1	*Augochlorella* sp. gut
*Apilactobacillus micheneri*	HV_65	GCA_006493595.1	*Megachile rotundata* (pollen)
*Apilactobacillus micheneri*	HV_23	GCA_006493625.1	*Dialictus* sp. gut
*Apilactobacillus timberlakei*	HV_25	GCA_006493105.1	*Dialictus* sp. gut
*Apilactobacillus timberlakei*	HV_12	GCA_002993965.1	*Augochlorella* sp. gut
*Apilactobacillus timberlakei*	HV_02	GCA_006493435.1	*Agapostemon* sp. gut
*Apilactobacillus timberlakei*	HV_26	GCA_006493425.1	*Agapostemon* sp. gut
*Apilactobacillus timberlakei*	HV_04	GCA_006493055.1	*Augochlorella* sp. gut
*Apilactobacillus timberlakei*	HV_10	GCA_006493095.1	*Augochlorella* sp. gut
*Apilactobacillus timberlakei*	HV_09	GCA_006493175.1	*Agapostemon* sp. gut
*Apilactobacillus timberlakei*	HV_28	GCA_006493125.1	*Agapostemon* sp. gut
*Apilactobacillus quenuiae*	HV_6	GCA_002994005.1	*Augochlorella* sp. gut
*Apilactobacillus apinorum*	Fhon13	GCA_946888465.1	*Apis mellifera*
*Apilactobacillus waqarii*	HBW1	GCA_019061205.1	*Apis mellifera*

**Table 2:** Genomes of *Ligilactobacillus salivarius* strains used in this work. The genomes were obtained from the National Center for Biotechnology Information (NCBI) database.

Species	Strain	GenBank	Isolation Source
*Ligilactobacillus salivarius*	A3iob	GCA_003129685.1	Honeybee intestine
*Ligilactobacillus salivarius*	ATCC11741	GCA_000159395.1	Human saliva
*Ligilactobacillus salivarius*	CECT5713	GCA_000143435.1	Feces of breast-fed human infant
*Ligilactobacillus salivarius*	CICC 23174	GCA_001723525.1	Chicken intestine
*Ligilactobacillus salivarius*	DJ-sa-01	GCA_003316955.1	Chicken intestine
*Ligilactobacillus salivarius*	FFIG58	GCA_013401855.1	Pig intestine
*Ligilactobacillus salivarius*	Gul1	GCA_002079565.1	Human saliva
*Ligilactobacillus salivarius*	Gul2	GCA_002079545.1	Human saliva
*Ligilactobacillus salivarius*	JCM1046	GCA_000758365.1	Pig intestine
*Ligilactobacillus salivarius*	LPM01	GCA_900094615.1	Human breast milk
*Ligilactobacillus salivarius*	REN	GCA_001011095.1	Human feces
*Ligilactobacillus salivarius*	TUCO-L2	GCA_004405135.1	*Lama glama* milk
*Ligilactobacillus salivarius*	UCC118	GCA_000008925.1	Human ileum
*Ligilactobacillus salivarius*	ZLS006	GCA_002162055.1	Pig intestine

## Data Availability

All relevant data are contained within the article: The original contributions presented in this study are included in the article/Supplementary Materials; further inquiries can be directed to the corresponding authors.

## Supplementary Materials

The following supporting information can be downloaded at https://www.mdpi.com/article/10.3390/ani15172606/s1. Supplementary Figure S1. Phylogeny analysis of Ligilactobacillus salivarius strains. (A) Phylogeny constructed with 16s RNA sequences. (B) MLST analysis using the genes parB, rpsB, pheS, nrdB, groEL, and ftsQ present in the genomes of *L. salivarius* strains. Colored boxes indicate the isolation source of each strain. Supplementary Figure S2. Distribution of glycosyl hydrolase (GH) (A) and glycosyl transferase (GT) (B) genes in *L. salivarius* A3iob genome compared with the genomes of strains of the same species. Colored boxes indicate the number of genes in each GH or GT family, or the isolation source of each strain. Supplementary Figure S3. Genes involved in metabolic pathways in *L. salivarius* A3iob genome. (A) Percentage of genes across different metabolic categories for *L. salivarius* A3iob compared with strains of the same species. (B) Number of genes across different metabolic categories for *L. salivarius* A3iob compared with strains of the same species. Supplementary Figure S4. Genes involved in the production of niacin (vitamin B3) (A) and pantothenic acid (vitamin B5) (B) in *L. salivarius* A3iob genome compared with the genomes of strains of the same species. The metabolic pathways are shown indicating the genes that encode the enzymes involved in each step. Colored boxes indicate the presence or absence of genes. Supplementary Figure S5. Genes involved in the production of thiamin (vitamin B1) in *L. salivarius* A3iob genome compared with the genomes of strains of the same species. The metabolic pathways are shown indicating the genes that encode the enzymes involved in each step. Colored boxes indicate the presence or absence of genes. Supplementary Figure S6. Genes involved in the production of biotin (vitamin B7) (A) and riboflavin (vitamin B2) (B) in *L. salivarius* A3iob genome compared with the genomes of strains of the same species. The metabolic pathways are shown indicating the genes that encode the enzymes involved in each step. Colored boxes indicate the presence or absence of genes. Supplementary Figure S7. Genes involved in the production of pyridoxine in *L. salivarius* A3iob genome compared with the genomes of strains of the same species. The metabolic pathways are shown indicating the genes that encode the enzymes involved in each step. Colored boxes indicate the presence or absence of genes. Supplementary Figure S8. Genes involved in the production of folate in *L. salivarius* A3iob genome compared with the genomes of strains of the same species. The metabolic pathways are shown indicating the genes that encode the enzymes involved in each step. Colored boxes indicate the presence or absence of genes. Supplementary Table S1. Genes involved in the resistance to antimicrobial compounds for bee related bacterial strains. Genomes of bacteria isolated from bee intestine, pollen or hive belonging to the genus *Apilactobacillus*, *Bombella*, *Lactobacillus*, *Bifidobacterium*, *Enterococcus*, and *Ligilactobacillus* were studied. Supplementary Table S2. Genes involved in virulence for bee related bacterial strains. Genomes of bacteria isolated from bee intestine, pollen or hive belonging to the genus *Apilactobacillus*, *Bombella*, *Lactobacillus*, *Bifidobacterium*, *Enterococcus*, and *Ligilactobacillus* were studied.
