# Supplementary material for: Genomic Characterization of the Honeybee–Probiotic Strain Ligilactobacillus salivarius A3iob
Source: Animals (Basel). 2025 Sep 5;15(17):2606. doi: 10.3390/ani15172606 (PMC12427466; doi:10.3390/ani15172606)
Supplement: Supplementary file 1 [file animals-15-02606-s001.zip › animals-3810125-supplementary.pdf]

animals

MDPI

*Supplementary Material*

# Genomic characterization of the honeybee-probiotic strain *Ligilactobacillus salivarius* A3iob

Mariano Elean[1,a], Alejandro Arroyo Guerra[2,a], Leonardo Albarracín[1], Keita Nishiyama[3,4], Haruki Kitazawa[3,4], Carina Audisio[2]*, Julio Villena[1]*

*** Correspondence:**
Corresponding Author Dr. Carina Audisio, e-mail: carina.audisio@gmail.com
Corresponding Author Dr. Julio Villena, e-mail: jcvillena@cerela.org.ar
Corresponding Author Dr. Haruki Kitazawa, e-mail: haruki.kitazawa.c7@tohoku.ac.jp

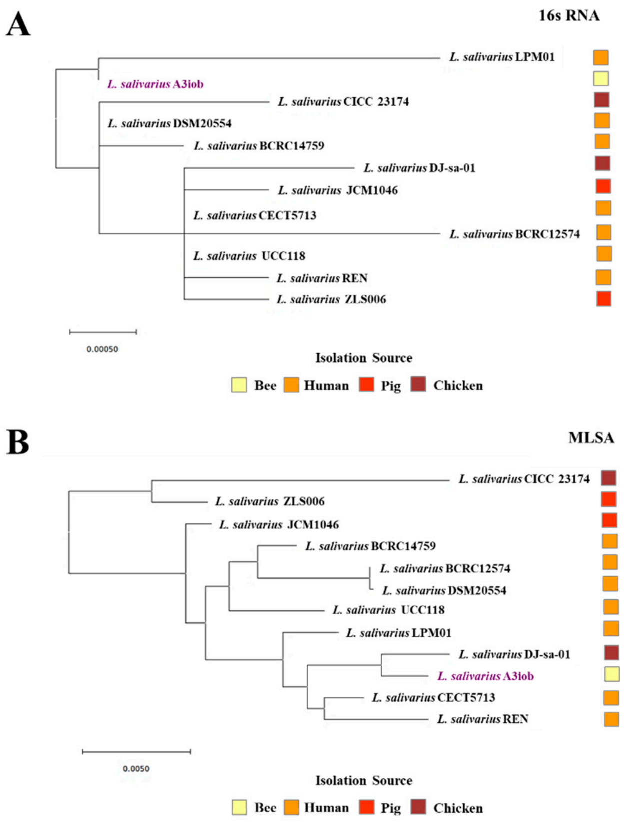


**Supplementary Figure S1.** Phylogeny analysis of *Ligilactobacillus salivarius* strains. (**A**) Phylogeny constructed with 16s RNA sequences. (**B**) MLST analysis using the genes *parB*, *rpsB*, *pheS*, *nrdB*, *groEL*, and *ftsQ* present in the genomes of *L. salivarius* strains. Colored boxes indicate the isolation source of each strain.

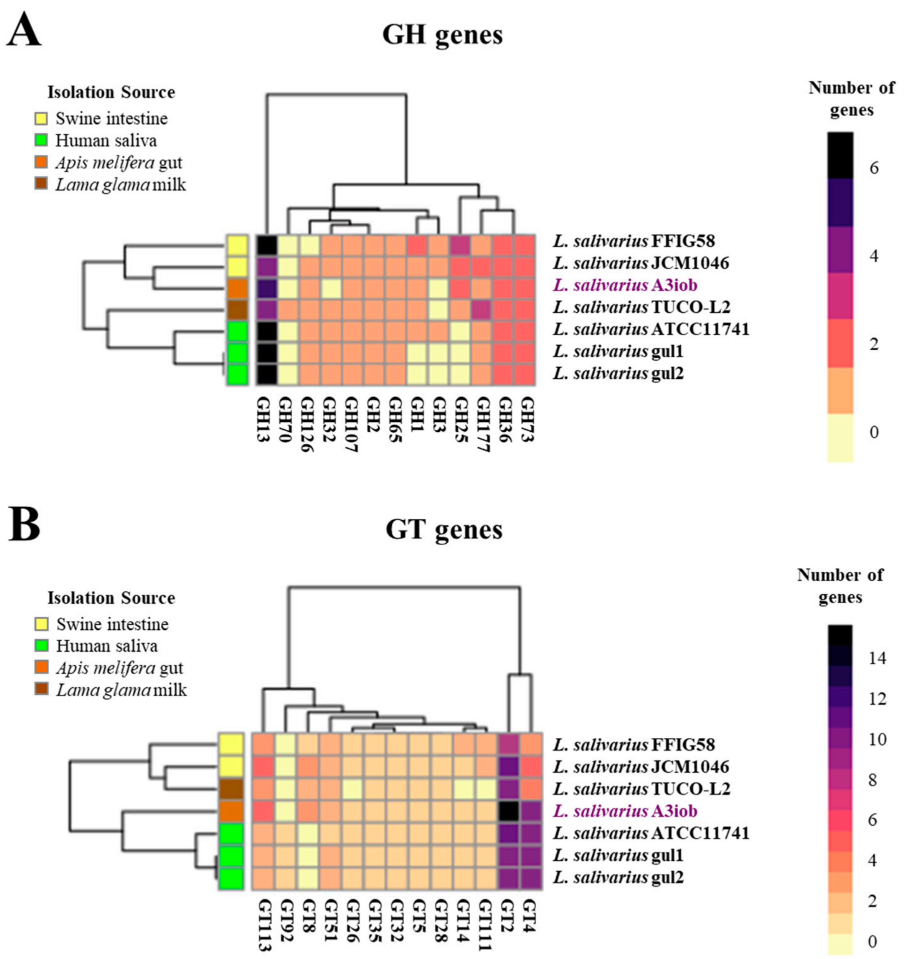


**Supplementary Figure S2.** Distribution of glycosyl hydrolase (GH) (**A**) and glycosyl transferase (GT) (**B**) genes in *Ligilactobacillus salivarius* A3iob genome compared with the genomes of strains of the same species. Colored boxes indicate the number of genes in each GH or GT family, or the isolation source of each strain.

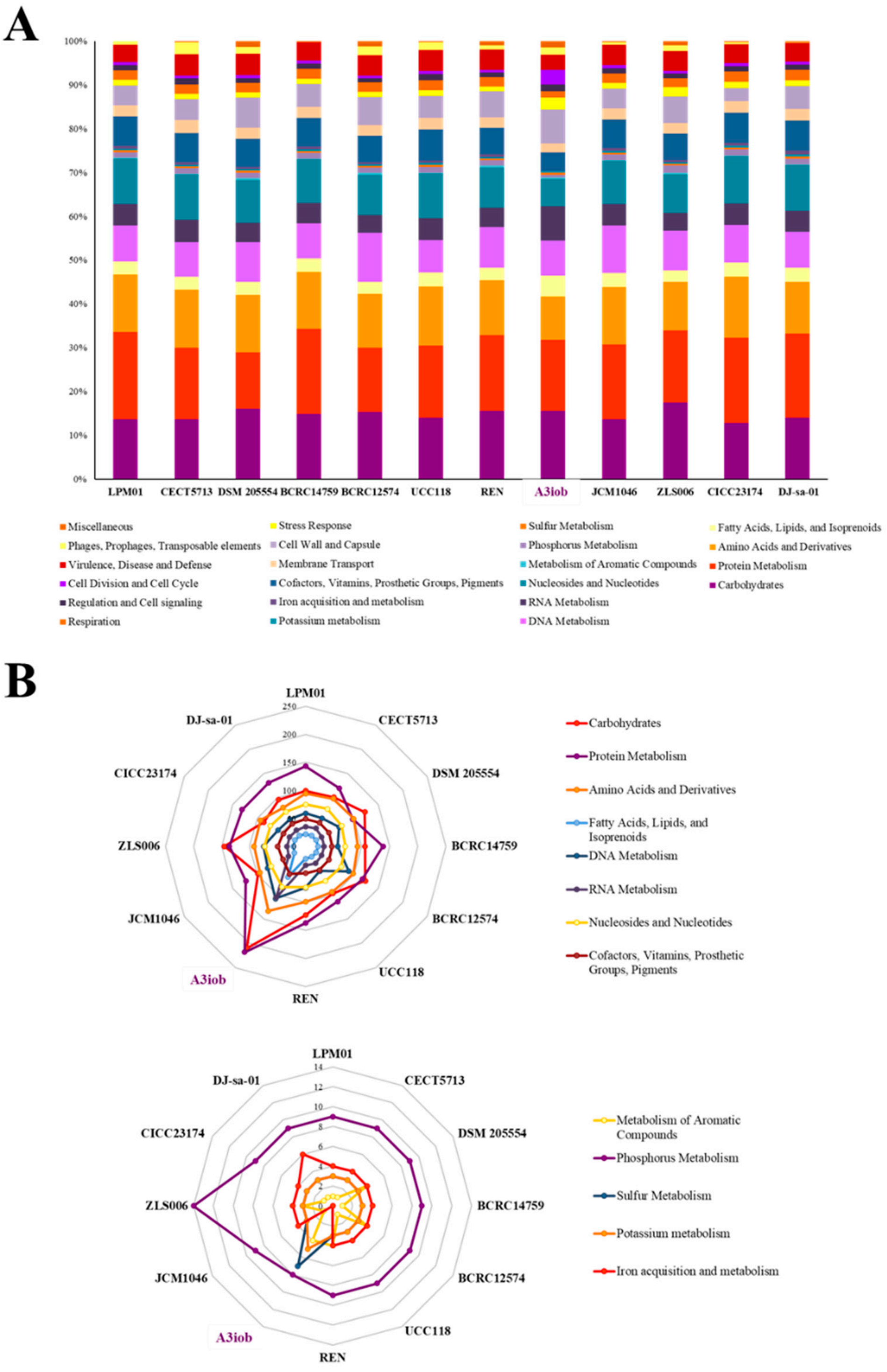


**Supplementary Figure S3.** Genes involved in metabolic pathways in *Ligilactobacillus salivarius* A3iob genome. (**A**) Percentage of genes across different metabolic categories for *L. salivarius* A3iob compared with strains of the same species. (**B**) Number of genes across different metabolic categories for *L. salivarius* A3iob compared with strains of the same species.

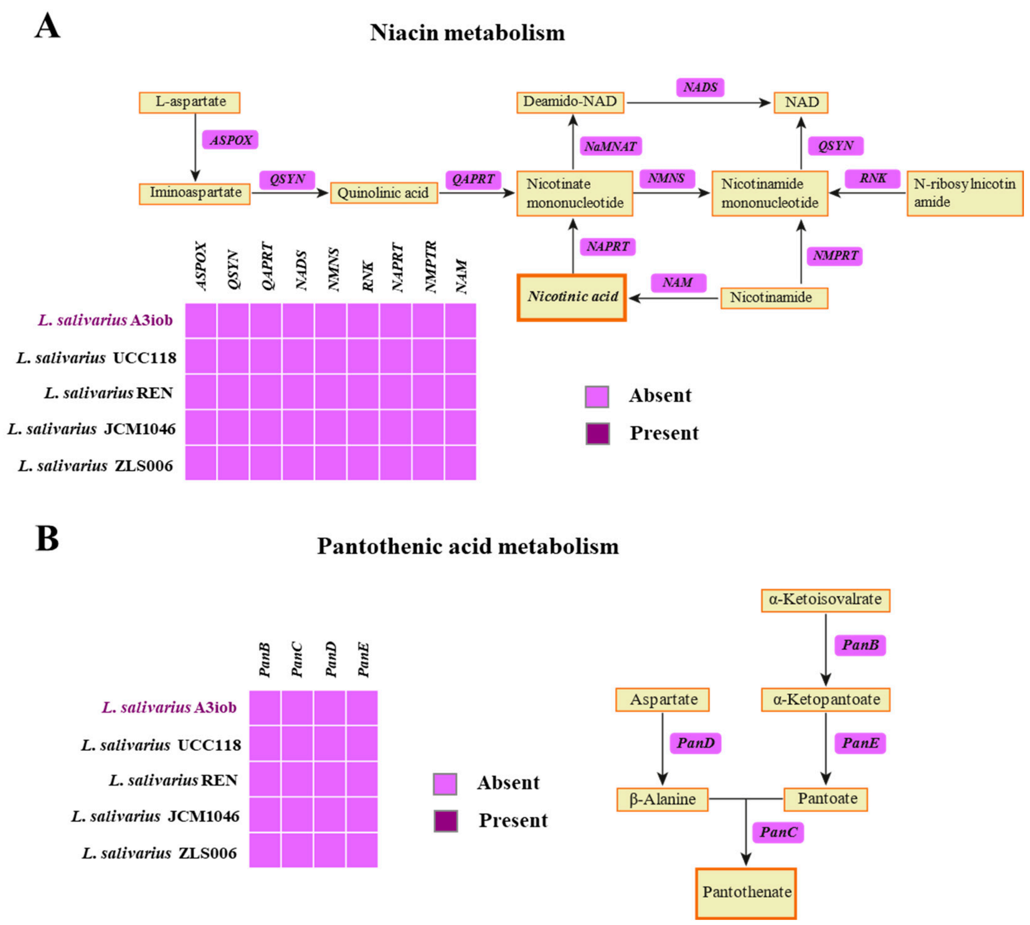


**Supplementary Figure S4.** Genes involved in the production of niacin (vitamin B3) (**A**) and pantothenic acid (vitamin B5) (**B**) in *Ligilactobacillus salivarius* A3iob genome compared with the genomes of strains of the same species. The metabolic pathways are shown indicating the genes that encode the enzymes involved in each step. Colored boxes indicate the presence or absence of genes.

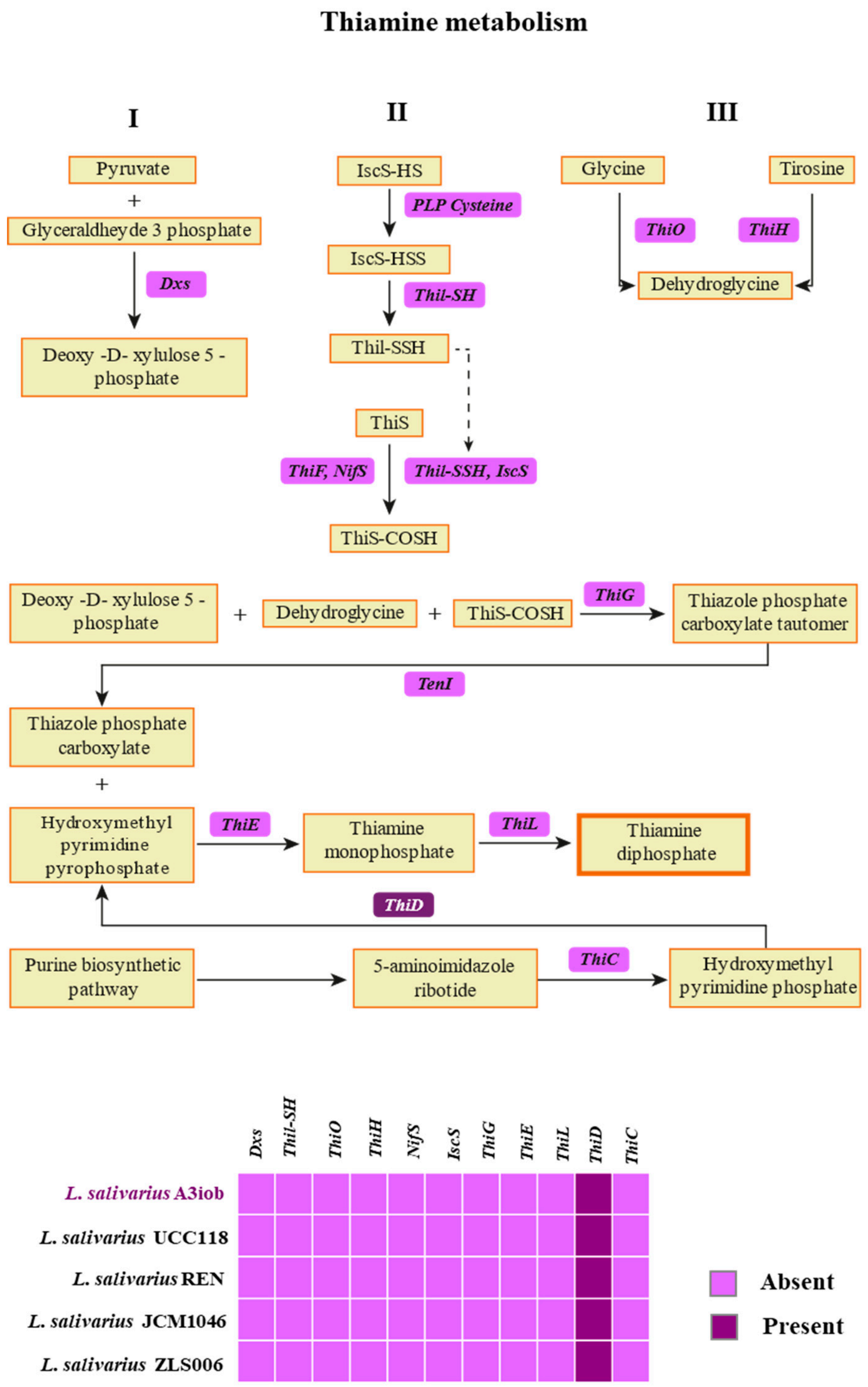


**Supplementary Figure S5**. Genes involved in the production of thiamin (vitamin B1) in *Ligilactobacillus salivarius* A3iob genome compared with the genomes of strains of the same species. The metabolic pathways are shown indicating the genes that encode the enzymes involved in each step. Colored boxes indicate the presence or absence of genes.

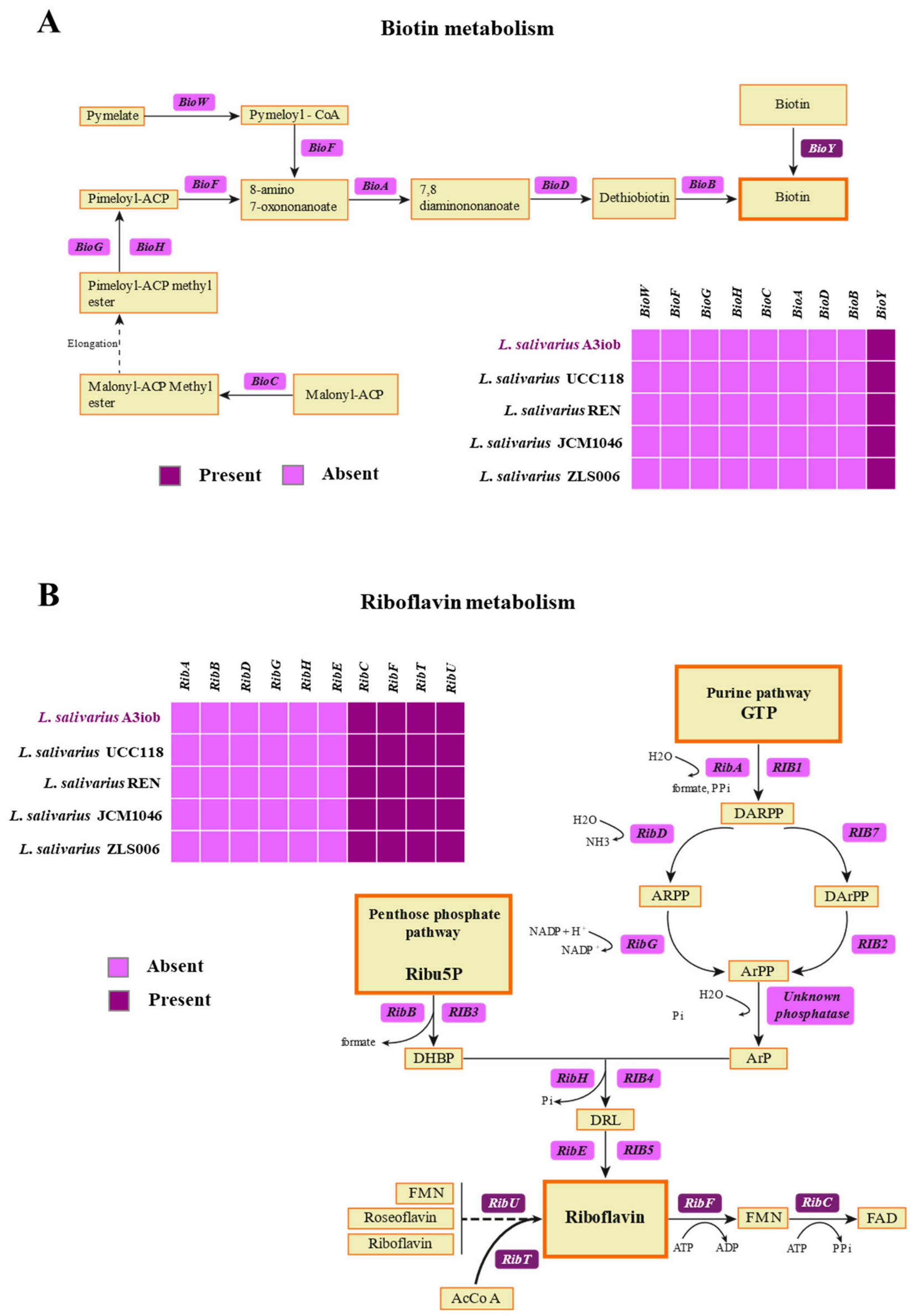


**Supplementary Figure S6**. Genes involved in the production of biotin (vitamin B7) (**A**) and riboflavin (vitamin B2) (**B**) in *Ligilactobacillus salivarius* A3iob genome compared with the genomes of strains of the same species. The metabolic pathways are shown indicating the genes that encode the enzymes involved in each step. Colored boxes indicate the presence or absence of genes.

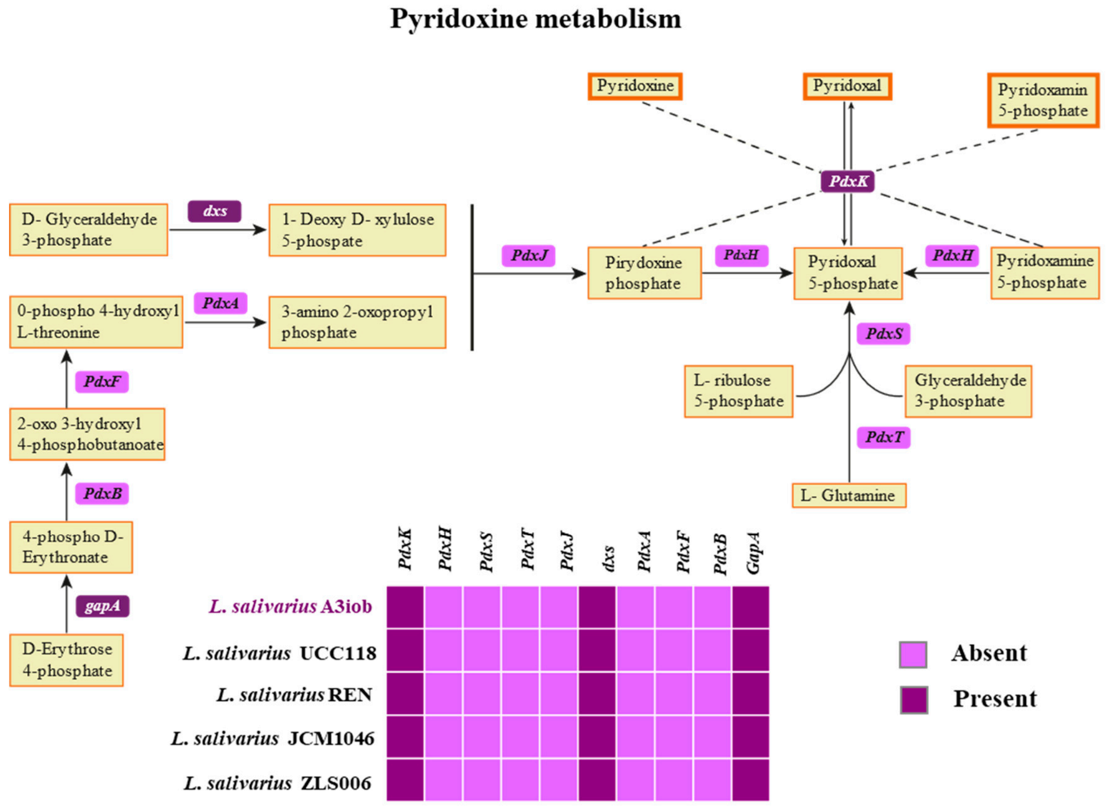


**Supplementary Figure S7.** Genes involved in the production of pyridoxine in *Ligilactobacillus salivarius* A3iob genome compared with the genomes of strains of the same species. The metabolic pathways are shown indicating the genes that encode the enzymes involved in each step. Colored boxes indicate the presence or absence of genes.

**Folic acid metabolism**

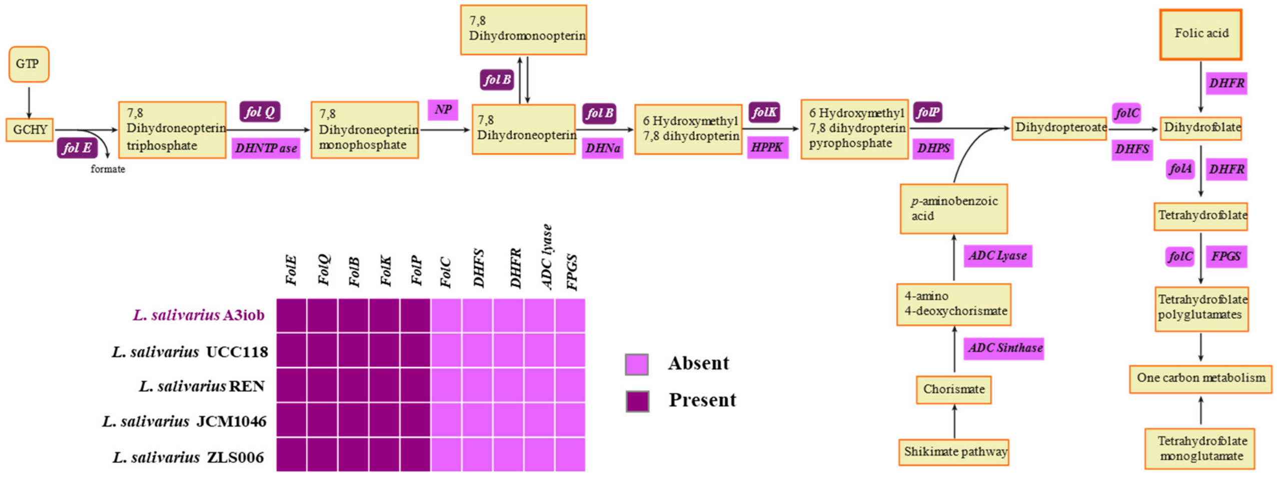


**Supplementary Figure S8.** Genes involved in the production of folate in *Ligilactobacillus salivarius* A3iob genome compared with the genomes of strains of the same species. The metabolic pathways are shown indicating the genes that encode the enzymes involved in each step. Colored boxes indicate the presence or absence of genes.

**Supplementary Table S1.** Genes involved in the resistance to antimicrobial compounds for bee related bacterial strains. Genomes of bacteria isolated from bee intestine, pollen or hive belonging to the genus *Apilactobacillus, Bombella*, *Lactobacillus*, *Bifidobacterium*, *Enterococcus*, and *Ligilactobacillus* were studied.

| Species | Strain | Gene | % identity matching region | % length of reference | Predicted by | |
|---|---|---|---|---|---|---|
| | | | | | RGI 6.0.3 | ResFinder-4.6.0 |
| Bifidobacterium sp. | 7101 | rifamycin-resistant beta-subunit of RNA polymerase (rpoB) | 89 | 100 | 1 | 0 |
| Bifidobacterium sp. | A11 | beta-subunit of RNA polymerase (rpoB) | 89 | 100 | 1 | 0 |
| Apilactobacillus kunkeei | AR114 | ErmB | 98 | 99 | 1 | 1 |
| Bifidobacterium animalis subsp. lactis | BI04 | tet(W) | 97 | 100 | 1 | 1 |
| | | beta-subunit of RNA polymerase (rpoB) | 93 | 100 | 1 | 0 |
| Enterococcus durans | EDD2 | AAC(6')-Iih | 100 | 100 | 1 | 1 |

**Supplementary Table S2.** Genes involved in virulence for bee related bacterial strains. Genomes of bacteria isolated from bee intestine, pollen or hive belonging to the genus *Apilactobacillus, Bombella*, *Lactobacillus*, *Bifidobacterium*, *Enterococcus*, and *Ligilactobacillus* were studied.

| Species | Strain | Code | Gene | Description | % identity | % similarity | Score | E-value |
|---|---|---|---|---|---|---|---|---|
| ***Bifidobacterium sp.*** | A11 | VFG047708 | carB | carbamoyl phosphate synthase large subunit | 48 | 68 | 1017 | 0 |
| ***Bifidobacterium sp.*** | 7101 | VFG048830 | gndA | NADP-dependent phosphogluconate dehydrogenase | 51 | 69 | 471 | e-131 |
| ***Enterococcus durans*** | EDD2 | VFG000964 | hasC | UTP--glucose-1-phosphate uridylyltransferase | 75 | 85 | 443 | e-123 |
